# SV-IV Peptide1–16 reduces coagulant power in normal Factor V and Factor V Leiden

**DOI:** 10.1186/1479-5876-5-69

**Published:** 2007-12-21

**Authors:** Biagio Di Micco, Marilena Lepretti, Lidia Rota, Ilaria Quaglia, Paola Ferrazzi, Gianluca Di Micco, Pierpaolo Di Micco

**Affiliations:** 1 University of Sannio Benevento Italy; 2 Department of Biochemistry and Biophysics, Second University of Naples, Italy; 3 Thrombosis Center, Istituto Clinico Humanitas, Milan, Italy; 4 Department of Internal Medicine, Buon Consiglio Fatebenefratelli Hospital of Naples, Italy; 5 Department of Biochemistry and Biotechnology and CEINGE, "Federico II" University of Naples, Italy

## Abstract

Native Factor V is an anticoagulant, but when activated by thrombin, Factor X or platelet proteases, it becomes a procoagulant. Due to these double properties, Factor V plays a crucial role in the regulation of coagulation/anticoagulation balance.

Factor V Leiden (FVL) disorder may lead to thrombophilia. Whether a reduction in the activation of Factor V or Factor V Leiden may correct the disposition to thrombophilia is unknown. Therefore we tested SV-IV Peptide 1–16 (i.e. a peptide derived by seminal protein vescicle number IV, SV-IV) to assess its capacity to inhibit the procoagulant activity of normal clotting factor V or Factor V Leiden (FVL). We found that SV-IV protein has potent anti-inflammatory and immunomodulatory properties and also exerts procoagulant activity. In the present work we show that the SV-IV Peptide 1–16, incubated with plasma containing normal Factor V or FVL plasma for 5 minutes reduces the procoagulant capacity of both substances. This is an anticoagulant effect whereas SV-IV protein is a procoagulant. This activity is effective both in terms of the coagulation tests, where coagulation times are increased, and in terms of biochemical tests conducted with purified molecules, where Factor X activation is reduced.

Peptide 1–16 was, in the pure molecule system, first incubated for 5 minutes with purified Factor V then it was added to the mix of phosphatidylserine, Ca2^+^, Factor X and its chromogenic molecule Chromozym X. We observed a more than 50% reduction in lysis of chromogenic molecule Chromozym X by Factor Xa, compared to the sample without Peptide 1–16. Such reduction in Chromozym X lysis, is explained with the reduced activation of Factor X by partial inactivation of Factor V by Peptide 1–16. Thus our study demonstrates that Peptide 1–16 reduces the coagulation capacity of Factor V and Factor V Leiden in vitro, and, in turn, causes factor X reduced activation.

## Introduction

We have recently studied the Protein SV-IV (Seminal vesicle protein number IV) [1-3]. According to its mobility in SDS/PAGE, SV-IV protein is a basic, thermostable, secretory protein of low molecular weight (9758 d) and it is synthesized by the epithelium of a rat seminal vesicle (SV) under strict androgen transcriptional control [1-4]. SV-IV immunorelated proteins have been found in several rat tissues (uterus, lung, liver, brain and so on), and in human seminal fluid secretion. The amino acids sequence of Protein SV-IV is 90 amino-acids long and is encoded by a gene that has been identified, sequenced and expressed in *Escherichia coli *[5-9].

We found that SV-IV Protein has potent anti-inflammatory and immunomodulatory properties and also exerts procoagulant activity [4,10-15]. We sought to find a correlation between a function of the molecule and its structure and to assess if portions of the protein, fragments or peptides, individually tested, would reveal one or more of the molecule's reported functions [4,10-15]. We already showed that the fragment 1–70 of SV-IV is responsible for the procoagulant function of the molecule [14-18]. We decided to study a small segments of such fragment 1–70 in order to assess which sequence was specifically responsible of its activity [4,17]. We synthesised small peptides such as SV-IV Peptide 1–8, Peptide 8–16 and Peptide 1–16 of the fragment 1–70 [4].

Factor V is synthesized by megacaryocites, endothelial cells and hepatocytes. Native Factor V is an anticoagulant, but when activated by thrombin, Factor X or platelet proteases, it becomes a procoagulant. Physiologically, it can be activated by Factor X activated (Xa) and platelet proteases but usually it is largely activated by thrombin, so Factor V becomes Factor V activated (Va) [19]. It is a large protein with a molecular weight of 300.000 d. Factor V binds the phospholipids of cellular membranes, such as platelets, endothelial cells, and monocytes, in the presence of calcium, and forms a receptor for Factor X and Prothrombin [20,21]. This last complex, named Prothrombinase, is responsible for cleaving prothrombin to form thrombin. This complex is much more efficient than Factor Xa alone in the prothrombin conversion. The native (i.e. inactivated) Factor V, however, is an anticoagulant. Native Factor V and Protein S are synergistic co-factors in the anticoagulant-inhibitory action of the activated Protein C [19,21], which inhibits coagulation by breaking down Factor Va (i.e. after its activation) and Factor VIIIa thus leading to a slow thrombin generation.

When activated, Factor V loses this anticoagulant property [19]. Consequently, due to its double properties in its native and activated forms, Factor V plays a relevant role in coagulation/anticoagulation balance [4,19]. Should Factor V anticoagulant capacity be utilised, it would be sufficient to inhibit or reduce the activation of Factor V.

Factor V Leiden (FVL) is a gene variant of clotting Factor V and it is responsible for the major forms of activated Protein C resistance. The structural difference of normal Factor V compared to Factor V Leiden consists in a substitution of an Arg with Gln in position 506, that is responsible for FVL absent lysis by the activated Protein C, but as far as factor X activation is concerned they are comparable [19-21]. The absent lysis in FVL causes thrombophilia by activating Factor X, therefore leading to an increase of the procoagulant potency, because normal Factor V is joined with FVL, that has not been broken down by activated Protein C. Therefore, we tried to reduce the procoagulant activity of normal Factor Va and also of FVL activated by Peptide1–16. Then, we also evaluated the activities of Peptide1–16 incubated with plasma containing normal Factor V or FVL in diversified coagulation systems in vitro and\or incubated with Factor V in a system of pure molecules.

## Methods

INBIOS Company, via Olivetti 1, Pozzuoli, Naples, Italy provided the organic synthesis on solid phase of SV-IV Peptide 1–16, since now is well known amino acid sequence of Protein SV-IV. The amino acids sequence is RKTKEKYSQSEEVVSE. The Thrombosis Center of the Istituto Clinico Humanitas, via Manzoni 56, Rozzano, Milan, Italy and the Department of Internal Medicine of the Buon Consiglio Fatebenefratelli Hospital of Naples provided plasma samples taken from twelve patients carriers of FVL.

Selected patients carriers of FVL were affected by previous thrombotic disease except 2 subjects that had not experienced any thrombotic manifestation in their anamnesis and that have been monitored for a couple of years.

Blood samples were obtained by venipuncture from antecubital vein and collected in sodium citrate 0.11 M tubes in 9:1 v\v in three aliquots of 5 mL and also in EDTA in one aliquot of 5 mL from all subjects.

All samples were tested to confirm the presence of FVL in order to confirm the thrombophilic state. The presence of FVL was confirmed with DNA extraction from the sample in EDTA and using the "NUCLEON BACC" kit (Amershan, Germany); Factor V G1691A gene polymorphism (i.e. FVL) was researched using PCR amplification with specific primers and the Light Cycler apparatus (Roche, Milan, Italy). Behring Institute (Protein C reagent, Dade Bhering, Milan, Italy) provided the kit to assess resistance to activated Protein C, which is altered in carrier of FVL as it contains Agkistrodom Contortix venom as the Protein C activator.

The activated partial thromboplastin time (aPTT) reagent (Ellagic acid and phospholipids) necessary for aPTT technique was also provided by Bhering Institute (Thromboplastin time, Milan, Italy).

Sigma, Milan, Italy, provided Factor V deficient plasmas, purified Factor V, purified Factor X, Phosphatidylserine and Chromozym X (Human Factor V deficient plasma, Sigma, Milan, Italy; Human Plasma Factor V, Sigma, Milan, Italy; Human Plasma Factor X, Sigma, Milan, Italy; Phosphatidylserine, Sigma, Milan, Italy; Chromozyn X, Sigma, Milan, Italy, respectively).

Once obtained all materials we developed study techniques to assess possible interference of Peptide 1–16 with Factor V and Factor V Leiden. We had to prove before the factor V activation to factor Va: in pure molecules factor V, phosphatidylserine, Ca2^+ ^and factor X system, we attempted to obtain factor X activation to factor Xa by factor V activation to Va; we have varied the physiological concentrations, increasing some component concentration of the system: phosphatidylserine, not phospholipid complex, and factor X increased concentration, because we have further coactivators lacking in vitro. After testing the 50 or 5 or 1 μM peptide1–16 concentractions both with plasmas and pure molecules we noted that 1 mM not produced variation whereas 50 and 5 μM produced same variations and both the concentrations required the incubation time of 5 minutes. Here we report the reagents used to perform the in vitro's experiences.

1) NaCl solution: 0,15 M

2) CaCl2 solution: 25 mM

3) aPTT reagent (phospholipids plus ellagic acid from the kit)

4) Peptide 1–16 solution: 50 μM

5) Factor V solution: 16,6 μM; concentration in tests 0,166 μM

6) Factor X solution: 61 μM;

7) Phosphatidylserine solution: 12,5 μg/μL

8) solution of Chromozym X substrate: 1,9 mM.

Experimental tests were performed by several study techniques:

 coagulative technique

 biochemical technique (conducted with purified molecules)

Coagulation techniques included the aPTT test technique, performed using factor V deficient plasma and purified Factor V; moreover a further coagulation technique was performed with Leiden plasmas compared to normal plasmas when incubated with Peptide 1–16 or NaCl as control.

A volume of 50 μl of aPTT reagent's was added to 100 μl of Factor V deficient plasmas and was incubated at 37°C for 1 minute and then 10 μl of purified Factor V incubated with 7 μl of Peptide 1–16 or 7 μl of NaCl for 5 minutes; then 100 μl CaCl2 was added and the coagulation time was recorded.

Coagulation techniques were also performed with Leiden plasma incubated for 5 minutes with Peptide 1–16 or NaCl; to 100 μl Leiden plasma plus 7 μl Peptide1–16 or NaCl incubated for 5 minutes was added 50 mcl aPTT reagent and, after incubation for 1 minutes, was furtherly added 100 μl CaCl2; then the coagulation time was registered. Thereafter the same procedure were performed with normal plasma: we incubated normal plasma for 5 minutes with Peptide 1–16 or NaCl; to 100 μl normal plasma plus 7 μl Peptide 1–16 or NaCl incubated for 5 minutes was added 50 μl aPTT reagent and after 1 minute, we added 100 μl of CaCl2, then the coagulation time was registered again. We chose to incubate the peptide 1–16 with purified factor V or normal or Leiden plasma for 5 minutes because the incubation for 5 or 7 or 9 minutes gave the same results in previous experiences (data not shown). Five tests were conducted on each plasma sample and the result was given as media with SD for all performed tests.

Biochemical techniques with pure molecules aimed to carry out Factor X activation by Factor Va. For Factor X activation, Factor V and FVL operate in the same way. Once activated, Factor X breaks down its chromogenic substrate Chromozym X. The scission of the Chromozym X produces a coloured molecule and its concentration and colour depended directly on the concentration of activated Factor X. The activation of Factor V to Va, that actives Factor X has been obtained with incubation of phosphatidylserine and Ca2^+^, and then added to Factor X.

The final concentrations of used molecules are shown below:

10 μl Phosphatidylserine (100 ng) and 10 μl of purified Factor V (0,02 μM) and CaCl2, then incubated at 37°C for 3 minutes. Then the process was: 10 μl Phosphatidylserine added to 10 μl of purified Factor V added to CaCl2 added to 20 μl of purified Factor X (12,2 μM) and incubated at 37°C for 3 minutes. Thereafter, a volume of 1,120 μl NaCl (0,15 mM) was added to 20 μl of Chromozym X. OD readings were taken at 405 nm after 15 minutes in order to know the Chromozym X lysis. Obtained values were recorded as control value of Chromozym X lysis.

We substitute Peptide 1–16 to NaCl and we tested the incubation of the mixture of purified Factor V with Peptide 1–16 for 5 minutes; this mix was then added to the phosphatidylserine, CaCl2 and factor X mixture and incubated for 3 minutes. Then, Chromozym X was added and OD readings were taken at 405 nm after 15 minutes incubation. Obtained values were recorded as results of the inhibiting clotting activities of peptide 1–16 against purified factor V.

Five tests were conducted on each samples. Result represent the media with SD of all performed tests.

### Statistical analysis

Statistical analysis was based on the one way analysis of variance (ANOVA); differences were considered to be significant if p was < 0.05. Statistical analysis were carried out using SPSS statistical software.

## Results

The coagulation time of aPTT test of normal plasmas and plasmas containing FVL and aPTT reagent plus NaCl and CaCl2 was normal and no differences were found between normal plasmas and Leiden plasmas: 36 +\- 3 seconds and 37 +/- 2 seconds respectively (data not shown). No differences were found between aPTT of normal plasmas and Leiden plasmas at time T0, when we performed the incubation with peptide 1–16 or NaCl (respectively 40 +/- 5 seconds and 38 +/- 3 seconds); when we incubated the Peptide 1–16 with normal or Leiden plasmas for 5 minutes, the coagulation time turned respectively to 96 +/- 6 seconds and 100 +\- 5 seconds reaching statistical significance (96 +/- 6 seconds versus 38 +/- 3 seconds, p: < 0.001, s; and 100 +/- 5 seconds versus 38 +/- 5 seconds, p: 0.001, s) (Table 1).

**Table 1:** aPTT coagulation time of normal plasma and Leiden plasma (100 μL) with and without incubation for 5 minutes with peptide 1–16 or NaCl as control.

**Plasmas incubated with peptide 1–16**	**aPTT coagulation time (sec.)**	**Plasmas incubated with NaCl**	**aPTT coagulation time (sec.)**	**p**
Normal plasma incubated at T0 with peptide 1–16	40 ± 5	Normal plasma incubated at T0 with NaCl	38 ± 3	0,16
Leiden plasma incubated at T0 with peptide 1–16	41 ± 4	Leiden plasma incubated at T0 with NaCl	40 ± 4	0,59
Normal plasma incubated with peptide 1–16 at T1	90 ± 6	Normal plasma incubated at T1 with NaCl	37 ± 4	0,000
Leiden plasma incubated at T1 with peptide 1–16	120 ± 9	Leiden plasma incubated at T1 with NaCl	38 ± 3	0,000

aPTT: activated partial thromboplastin time

sec.: seconds

T0: time of incubation

T1: 5 minutes after the incubation

NaCl: sodium clorure

Moreover, the coagulation time was also obtained by the aPTT test with deficient Factor V plasma and purified Factor V incubated with NaCl or Peptide 1–16 for 5 minutes plus aPTT reagent (Ellagic acid and phospholipids) and CaCl2. When we incubated NaCl, the coagulation time was normal: 35 +\- 4 seconds and 39 +/- 4 seconds respectively; when the Peptide 1–16 substituted NaCl, the coagulation time was significantly longer: 96 +\- 7 seconds, p: < 0.001 (Table 2).

**Table 2:** aPTT coagulation time of deficient factor V plasma added to purified factor V (100 μL) and incubated with peptide 1–16 or NaCl as control.

**Test**	**aPTT coagulation time (sec.)**	**Test**	**aPTT coagulation time (sec.)**	**p**
Deficient factor V plasma added to purified factor V and NaCl	36 ± 4	Deficient factor V plasma added to purified and peptide 1–16	119 ± 7	0,000

aPTT: activated partial thromboplastin time

sec.: seconds

NaCl: sodium clorure

As control of previous experiments, in the biochemical tests, purified Factor V activation was obtained by the incubation for 3 minutes with phosphatidilserine, CaCl2, in presence of factor X with Chromozym X, and NaCl (as control of next tests) in order to activate Factor X with pure molecules: the Factor X incubated for 3 minutes and Chromozym X, incubated for 15 minutes. We obtained a coloured product with an optical density (O.D.) of 0.420 nm. On the other hand, when we incubated Factor V with Peptide1–16 (instead of NaCl) for 5 minutes and then added to the mixture of phospatidilserine and CaCl2 with subsequent addition of factor X and Chromozym X, we obtained a significantly different optical density (O.D.) of 0.190 nm, p: < 0.0001, s (Table 3).

**Table 3:** Factor X activation test (O.D.): purified factor V (0.002 μM) incubated with peptide 1–16 or NaCl for 5 minutes and thereafter added to phosphatidilserine and CaCl2 and factor X and Chromozyn X for 15 minutes.

**Parameter**	**Mixture with NaCl**	**Mixture with peptide 1–16**	**p**
O.D. (nm) of Chromozyn X	0.420 ± 0.008	0.190 ± 0.006	0,000

O.D.: optical density

nm: nanometers

CaCl2: calcium clorure

NaCl: sodium clorure

## Discussion

Whether factor V has sufficient endogenic activity to allow small amount factor Xa generation is still a matter of debate [22]. Data from literature report that factor V circulates as a single chain protein with about 0.27% of proteolitic activity comparable to Va [23]. Foster *et al.*[24] postulate that factor V appears to be a procofactor with 1/400 the activity of full activated factor V (i.e. factor Va). The proteolytic conversion of factor V to factor Va is catalysed by thrombin and Xa. However, because factor Va is required for thrombin generation, the initial participation of factor V in the expression of prothrombinase activity is not well understood [24]. Orfeo et al. demonstrated [25] that prothrombinase complex consists of the protease factor Xa, Ca2 and factor V assembled on phospholipid membrane. Factor Va functions both as a receptor for factor Xa and as a positive effector for factor Xa catalytic efficiency, thus being a key factor for an efficient conversion of prothrombin to thrombin. The activation of the procofactor factor V to factor Va is an essential reaction that occurs early in the process of tissue factor pathway, however the catalytic sequence leading to formation of factor Va is still a controversial issue [25]. We attempted, by a system consisting of pure molecules (i.e. factor V, phosphatidylserine, Ca2 and factor X) to obtain factor X activation to Xa through factor V activation to factor Va. We varied the physiological concentrations, increasing the same components of the system and chose to work only with phosphatidylserine and not phospholipid complex and to increase factor X concentration. This system produced factor X activation to Xa. We tested factor Xa peptidolytic activity with Chromozym X, that is a specific chromogenic molecule of factor Xa; when we added Chromozym X to this system, it generated colour, indicating that factor X in our system became Xa. Then, we incubated purified factor V with Peptide 1–16 for 5 minutes, and thereafter we added it to the same mixture, in order to prove if this Peptide 1–16 hampers factor V activation. In this system, in fact, factor X activation is reduced, as demonstrated by reduced lysis of chromozym X.

FVL is an inherited condition leading to thrombophilia through the activated protein C resistance. In presence of FVL, the activated Protein C does not breakdown FVL, that accumulates so increasing the global coagulative power in the coagulative cascade and impairing the coagulation\anticoagulation balance. The results of our experiments demonstrate clearly that Peptide1–16 inhibits the activity both of FVL and of normal clotting factor V, in particular its ability to activate Factor X. We demonstrated that the Leiden Plasmas incubated with the Peptide 1–16 cause an increase in the coagulation time in the test of aPTT, as reported in Table 1.

Data obtained by incubating Peptide 1–16 with purified Factor V for 5 minutes, then added to factor V deficient plasma, confirmed such results. In fact when we added this mixture to deficient Factor V Plasmas in an aPTT test we found a prolonged coagulation time compared to control (i.e. with NaCl without peptide 1–16), as reported in Table 2.

Moreover, considering that in the Factor X activation, Factor V and FVL operate in the same way, to verify Factor X activation, we only tested the ability of purified Factor V activated by pure molecules as phosphatidilserine and CaCl2. Then we added this mixture to Factor X and Chromozym X, in order to cause the lysis of Chromozym X by Xa: we obtained an optical density of 0.420 nm which clearly demonstrates the lysis of Chromozym X. Yet, if we incubate for 5 minutes Factor V and Peptide 1–16 and we add them to the mixture of pure molecules (phosphatidilserine and CaCl2) and thereafter to Factor X and Chromozym X, we observed that Factor X activation caused a reduced lysis of Chromozym at an optical density of 0,190 nm. Therefore, Factor X activation is significantly slower compared to control (i.e. NaCl and purified Factor V), and this is confirmed by a reduced lysis of Chromozym X (i.e. O.D. = 0.190).

The occurring contact of the two molecules, Factor V and Peptide1–16, is proved by the fact that both incubated at time 0 and added to phosphatidylserine and CaCl2 produced a different effect compared to that obtained if we incubate Factor V, alone for 5 minutes, without Peptide 1–16. This suggests that the observed events were caused by the incubation of the two molecules and that 5 minutes are needed for their effective interaction. Our results indicate that Peptide 1–16 is not only an inhibitor of normal clotting factor V but also of FVL. So, Peptide 1–16 may represent an inhibitor of clotting cascade also in presence of inherited thrombophilic disorder leading to hypercoagulable state as FVL. We demonstrated, in fact, the inhibition of clotting cascade in presence of normal factor V and also in the presence of plasmas containing FVL by both coagulation techniques and\or biochemical approaches.

One may argue that in our studies we used purified factor V to test the normal cascade and FVL from plasma of subjects carrying this kind of alteration. However we point out that in biochemical tests, normal purified factor V alone was incubated with Peptide 1–16 while purified FVL was not incubated with Peptide 1–16 because purified FVL is not available commercially both for experimental studies and\or clinical approaches. Moreover it is really difficult to obtain purified FVL from plasma of subjects carrying this alteration. Yet, the presence of purified FVL is not necessary for our studies because the role of normal Factor V and Factor V Leiden in Factor X activation is comparable.

In conclusion, our study demonstrates that Peptide1–16 may represent a specific antagonist of FVL and one may envision its use in the thrombophilic FVL patients, should further studies confirm our results.

## Competing interests

The authors declare that they have no competing interests, nor any financial supports during patients' selection, experimental tests and article extension.

## Authors' contributions

BDM and ML performed all biochemical tests described in the paper with the exception of genetic tests for factor V Leiden gene variant; IQ and PF performed genetic test to identify factor V Leiden gene variant; LR, GDM and PDM performed patients' selection; PDM and BDM performed paper extension.
